# A comparison of dietary isotopes in pulp stones and incremental dentine from Early Neolithic individuals of the Whitwell Long Cairn, England

**DOI:** 10.1002/ajpa.24479

**Published:** 2022-01-30

**Authors:** Brett Ostrum, Darren R. Gröcke, Janet Montgomery

**Affiliations:** ^1^ Department of Archaeology Durham University Durham UK; ^2^ Department of Earth Sciences Durham University Durham UK

**Keywords:** incremental dentine, Neolithic, palaeodiet, pulp stones, stable isotope analysis

## Abstract

**Objectives:**

This study investigates if palaeodietary information can be obtained from pulp stones through stable isotope analysis, presents a method for their extraction from tooth samples, and assesses their utility as a source of paleodietary information when coupled with the incremental dentine method.

**Materials and Methods:**

Six tooth samples (2 per individual), four of which contained pulp stones, were selected from three Early Neolithic (3720–3650 cal BC) individuals from the Whitwell Long Cairn in Derbyshire, England. After demineralization, each tooth was divided into 1 mm increments. Stable isotope analysis of collagen was conducted on each dentine increment and a portion of each pulp stone.

**Results:**

All samples met the quality control criteria for well‐preserved collagen. Excluding the pulp stones, the mean δ^13^C value of the teeth sampled was −21.5 ± 0.2‰ and the mean δ^15^N value was 9.9 ± 0.5‰, suggesting these individuals had a terrestrial‐based diet. The pulp stones produced similar δ^13^C values between −21.6 and −21.4‰ and δ^15^N values between 9.1 and 9.8‰.

**Discussion:**

The results demonstrate that paleodietary information can be obtained from pulp stones through stable isotope analysis. There are, however, significant challenges in interpreting this data, particularly as to inferring the timing and duration of their formation. The pulp stone results were compared with the incremental dentine profiles for each person to further investigate when they might have formed. For two individuals, the pulp stones appear to reflect diet from a time period after childhood and adolescence. For the third individual, it could not be determined if the pulp stones reflect a contemporary or later time period than the incremental dentine series. All teeth with pulp stones have moderate to severe wear on the occlusal surface, which could have been a contributing factor to their development.

## 1 INTRODUCTION

Pulp stones are mineralized masses that can form within the dental pulp of both deciduous and permanent teeth (Goga et al., 2008). Research on pulp stones has been dominated by endodontic studies, which have tended to focus on their prevalence, morphology, and possible etiologies in modern groups. The potential of pulp stones to provide information about past populations, however, is still largely unknown (Legge & Hardin, 2015). The present study seeks to investigate if stable isotope analysis of pulp stones can be used to examine the diets of three Early Neolithic individuals from the Whitwell Long Cairn. During incremental dentine sampling for a larger isotopic research study on this skeletal assemblage, we found that multiple individuals had pulp stones in one or more of their mandibular molars. In our experience sectioning human teeth for isotope analysis, this is an extremely uncommon finding in archeological humans from Britain. Moreover, to our knowledge no studies have yet undertaken stable isotope analysis of pulp stones to investigate their potential for palaeodietary reconstructions. This preliminary study presents stable carbon (δ^13^C) and nitrogen (δ^15^N) isotope results of collagen from pulp stones and incremental dentine with the goal of communicating about pulp stones to *AJBA* readers, including describing a method for their removal and analysis, and assessing their utility as a source of isotopic dietary information.

### 1.1 Pulp stones

**FIGURE 1:**
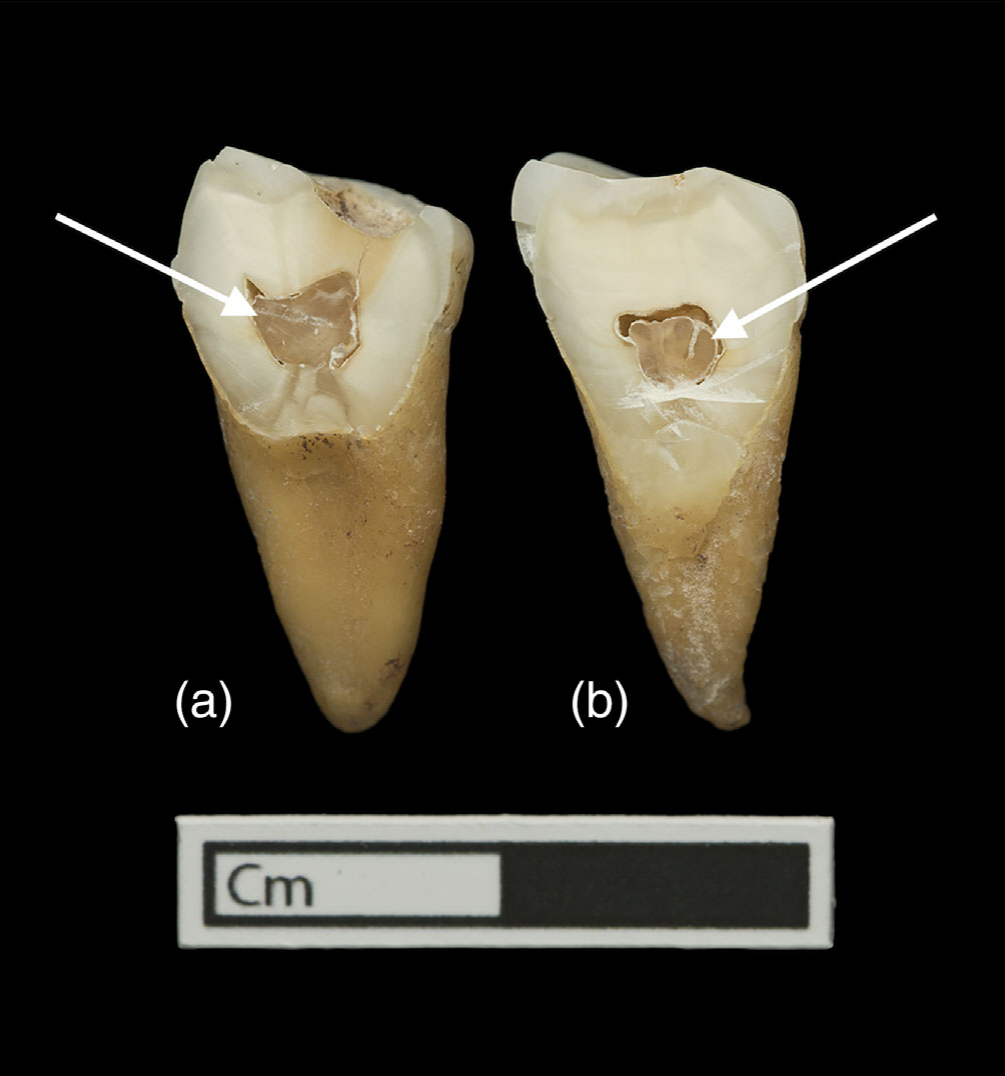
Teeth from an Early Neolithic individual (WHIT 0334) from the Whitwell Long Cairn. Arrows point to the pulp stones in each tooth. (a) Left first mandibular molar (distal half) and (b) left second mandibular molar (distal half)

Pulp stones begin forming as small, free‐floating mineralized objects within the dental pulp, and grow in size as continuing mineralization builds up on their outer edges in concentric layers (Moss‐Salentijn & Hendricks‐Klyvert, 1988). There may be multiple such masses within the pulp chamber of a single tooth, which can attach to one another and to the chamber walls, resulting in some pulp stones eventually growing large enough to completely occlude the pulp chamber (Hillson, 1996; Figure 1). Pulp stones can occur in all types of teeth, but are most prevalent in molars, especially in the maxillae (Legge & Hardin, 2015).

Pulp stones are sometimes referred to as “denticles” within the literature, but a number of researchers suggest this particular term be reserved for a possibly different type of calcified material that occurs within the radicular pulp, which are thought to originate from epithelial cell remnants or detached strands rather than from a calcified node (Hillmann & Geurtsen, 1997; Moss‐Salentijn & Hendricks‐Klyvert, 1988; Moss‐Salentijn & Klyvert, 1983). These calcified objects have a distinctive thimble‐shaped appearance, but can develop further into an enclosed shell with a noncalcified central cavity, whereas pulp stones have a solid core (Moss‐Salentijn & Klyvert, 1983). Pulp stones have similarly been categorized as “true” or “false” in some past studies based on morphological traits. True pulp stones were thought to be composed of dentine, whereas false pulp stones were thought to be pulp cells that had degenerated and become mineralized (Goga et al., 2008). The two types were thought to be distinguishable by the presence or absence of tubular dentine respectively, though this distinction is generally no longer considered useful or accurate as most calcified pulp material contains both tubular and atubular dentine (Goga et al., 2008; Hillmann & Geurtsen, 1997; Le May & Kaqueler, 1991; Moss‐Salentijn & Hendricks‐Klyvert, 1988; Moss‐Salentijn & Klyvert, 1983).

Structurally, pulp stones consist of collagen and bioapatite. They vary somewhat in their morphology, both in composition and in appearance. Differing amounts of orthodentine, fibrodentine, and calcified tissue have been found between pulp stones, and variations in their shape and surface texture are often observed (Berès et al., 2016; Le May & Kaqueler, 1991; Milcent et al., 2019; Moss‐Salentijn & Hendricks‐Klyvert, 1988; Ninomiya et al., 2001). Immunohistochemical studies have identified type I collagen as a major component of the organic matrix of pulp stones, and noted that it is evenly distributed throughout the entire pulp stone structure (Hillmann & Geurtsen, 1997; Ninomiya et al., 2001). Type III and VI collagen have also been identified (Hillmann & Geurtsen, 1997). The mineral component of pulp stones primarily consists of calcium and phosphorous (Le May & Kaqueler, 1993), with the crystal structures of the bioapatite being very similarly arranged to that of dentine (Berès et al., 2016; Milcent et al., 2019). Beyond this, pulp stones display heterogeneity in their chemical signature, with elements such as sodium, potassium, iron, copper, zinc, strontium, and magnesium found in varying amounts between pulp stones, even in those from the same individual (Berès et al., 2016; Milcent et al., 2019). Bioapatite from the inside region of pulp stones is chemically similar to dentine, while bioapatite from the outer edges tends to have a greater variety of chemical elements than typically found in dentine (Milcent et al., 2019). This difference may be from contact between pulp stones and organic tissues within the pulp chamber (Milcent et al., 2019).

The underlying cause of pulp stones is unknown (Goga et al., 2008), but it appears they may have multiple etiologies. In some cases, their formation is likely triggered by irritation and inflammation of the dental pulp, such as from advanced dental wear, severe caries, and dental restorations (Şener et al., 2009; Tomczyk et al., 2017). Clinical studies have also noted there may be a connection between pulp stone prevalence and certain genetic diseases like dentin dysplasia (VanDenBerghe et al., 1999). Edds et al. (2005) found a correlation between pulp stones and cardiovascular disease, though this has not always been found in subsequent research (e.g., Şener et al., 2009). Several studies have found a correlation between age and pulp stone frequency (e.g., Çolak et al., 2012; Gulsahi et al., 2009; Tomczyk et al., 2014), though others have found no such correlation (e.g., Hamasha & Darwazeh, 1998; Kannan et al., 2015; Tomczyk et al., 2017). Although few studies on pulp stones have been conducted on archaeological and historical human remains, osteological studies of pulp stones in skeletal assemblages from Australia (Elvery et al., 1998), Syria (Tomczyk et al., 2014), Poland (Tomczyk et al., 2017, 2020), and Germany (Nicklisch et al., 2021) have all found positive correlations between the occurrence of pulp stones and progressive dental wear.

Recent bioarcheological studies have begun exploring the possibility of a link between dietary practices and pulp stone prevalence. Tomczyk et al. (2014) sought to examine diet and pulp stone frequency by using spectrophotometry to evaluate elemental concentrations such as barium, calcium, and strontium in pulp stones from two multi‐period skeletal assemblages from Syria. The diets of these populations were thought to have varied over time, particularly in the level of dairy products consumed. Although Tomczyk et al. (2014) did not find any statistically significant differences in pulp stone prevalence across the time periods examined, they proposed that diets high in calcium, such as from the consumption of dairy products, could be linked to an increase in pulp stone frequency. Nicklisch et al. (2021) have also recently examined a potential link between pulp stone formation and diet by comparing stable nitrogen isotope ratios of rib samples from individuals with pulp stones to those of individuals without pulp stones from a number of archeological and historical skeletal assemblages from Germany. They found that individuals with pulp stones had a higher group mean δ^15^N value than the individuals without pulp stones, which they suggest tentatively supports the idea that pulp stone prevalence may have a positive correlation with diets high in animal protein (Nicklisch et al., 2021).

### 1.2 Stable isotope analysis of incremental dentine

Stable isotope ratios of carbon (δ^13^C) and nitrogen (δ^15^N) can provide a substantial amount of information about an individual's diet and the environments from which they acquired their food. δ^13^C and δ^15^N values vary with trophic level within a food web (Bocherens & Drucker, 2003) and, when used in conjunction, can determine if a person was consuming animals and plants from terrestrial and/or marine ecosystems (Schoeninger et al., 1983). Stable isotope analysis of incremental dentine, in which a tooth is divided into a series of small sections from crown to root, produces a dietary profile with high temporal resolution corresponding to early childhood through early adulthood depending on the specific teeth analyzed (Beaumont et al., 2013; Beaumont & Montgomery, 2015). Unlike bone collagen, which reflects an average of an individual's diet over a number of years because bone continuously remodels throughout life (Hedges et al., 2007), collagen from primary dentine will reflect diet during the period of tooth formation as primary dentine does not remodel (Beaumont et al., 2013). This temporal quality makes the incremental dentine method capable of identifying short‐term dietary changes (Montgomery et al., 2013), and periods of nutritional stress (Beaumont & Montgomery, 2016).

In this study, it is assumed that the δ^13^C and δ^15^N values of the pulp stones will represent diet over the entire course of their formation. As such, the isotopic results from the pulp stones will likely reflect a person's average diet over longer periods of time than the values of the dentine increments. Since the organic matrix of dentine is predominantly composed of type I collagen (Nanci, 2018), which is likewise a major component of the organic matrix of pulp stones (Ninomiya et al., 2001), it is assumed here that the amino acid profiles of the two dental tissues are similar and the isotopic results comparable.

### 1.3 Aims and objectives

To meet the goal of communicating about the information we obtained from pulp stones and dietary isotopes, the present study has the following aims and objectives:

1. To determine if pulp stones can provide palaeodietary information through carbon and nitrogen stable isotope analysis.
2. To present a method for removing and analyzing pulp stones that can be easily replicated.
3. To assess the utility of pulp stones as a source of palaeodietary information by considering factors that complicate interpretations of the results, including the uncertain timing of their formation, the influence of health status on δ^13^C and δ^15^N values, and possible etiologies that initiated their development.

## 2 MATERIALS AND METHODS

### 2.1 The Whitwell Long Cairn

**FIGURE 2:**
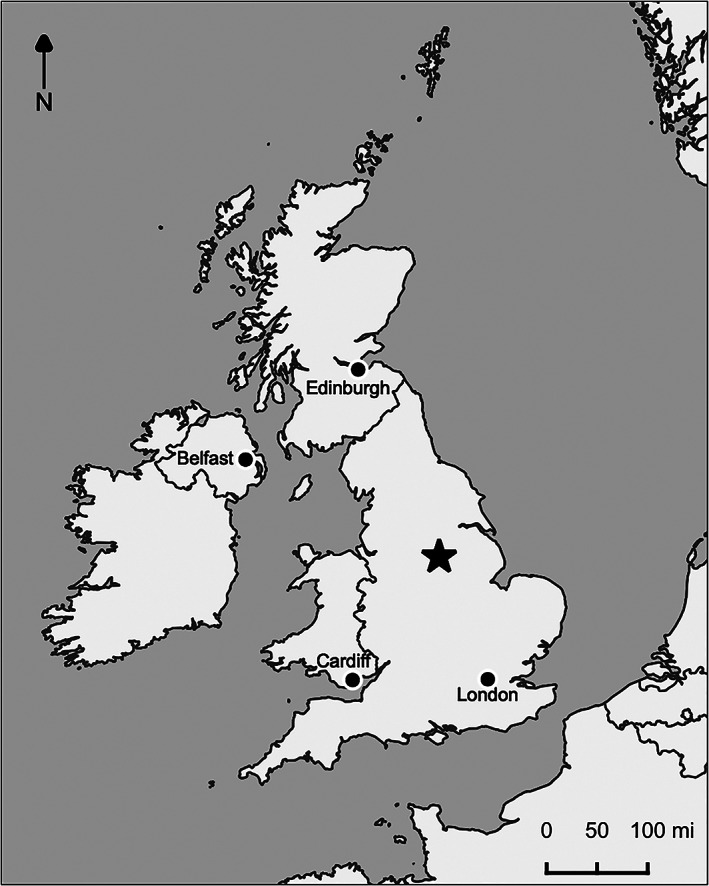
Map of the United Kingdom with a star showing the location of the Whitwell Long Cairn

Located near the village of Whitwell in Derbyshire, England (Figure 2), the Whitwell Long Cairn was in use from 3790–3710 cal BC to 3630–3540 cal BC (Vyner & Wall, 2011). The cairn was discovered in 1988 during an archeological survey of a modern limestone quarry and was completely excavated in 1988–1991 (Vyner & Wall, 2011). These excavations revealed a complex monument with multiple phases of construction and remodeling. Human remains were found in two contexts within the monument: a linear feature with comingled remains, and a single inhumation located beneath an earlier oval cairn. These two contexts were originally separate features, but were incorporated beneath a single trapezoidal cairn at a later date in the monument's history (Vyner & Wall, 2011).

During its earliest phase, the monument consisted solely of a linear mortuary structure, which was set between two wooden posts and likely had side walls of timber or wattle (Vyner & Wall, 2011). A minimum of five individuals, four adults and one nonadult, were deposited during this phase between 3790 and 3710 cal BC (95% probability) (Chamberlain & Witkin, 2011; Vyner & Wall, 2011). In the next phase of the monument, a single inhumation was deposited 7 meters south of the linear structure, which was later covered with an oval cairn. A trapezoidal cairn was then built over both the oval cairn and the linear structure with a passage for continued access to the linear feature (Vyner & Wall, 2011). A second period of mortuary activity occurred within the linear feature, with a minimum of five adults and one child deposited during this phase between 3720 and 3650 cal BC (95% probability) (Vyner & Wall, 2011). At an unknown date, the passage was sealed with limestone slabs at its juncture with the linear feature, and further stones were piled across the outside entrance and wall of the cairn (Vyner & Wall, 2011). With this sealing of the monument, Neolithic activity appears to have ended at the site.

More than 900 disarticulated skeletal elements and bone fragments were recovered from the linear mortuary feature during the excavation. These skeletal remains were highly fragmented and in poor condition, probably in part due to repeated access of the linear mortuary feature during its use, but acidic soil conditions and the collapse of the feature structure likely also contributed to the poor state of this skeletal assemblage (Chamberlain & Witkin, 2011). Because of their fragile condition, a polyvinyl acetate (PVA) consolidant was applied to many of the skeletal remains during the excavation to aid in their recovery (Vyner & Wall, 2011). This type of consolidant is removable with organic solvents, such as acetone, and has been shown to not interfere with stable isotope analysis of collagen when removed from bone samples (France et al., 2011). There is no nitrogen within this type of consolidant, so there is no possibility of this particular element being altered. While the consolidant does contain carbon, PVA stock pellets have been recorded by France et al. (2011) as having δ^13^C values around −31.4‰. This δ^13^C value is much lower than any expected δ^13^C values from human collagen, which France et al. (2011) have suggested provides a tracer in itself for any possible contamination from PVA consolidants in δ^13^C results.

### 2.2 Sample selection

Three individuals from the Whitwell Long Cairn skeletal assemblage that were discovered to have pulp stones in one or more of their mandibular molars were selected for the present study. Two teeth from each of these individuals were sampled for stable isotope analysis (Table 1). All three individuals were interred within the linear feature during the second period of mortuary activity (3720–3650 cal BC).

**TABLE 1:** Individuals from the Whitwell Long Cairn selected for stable isotope analysis of incremental dentine and pulp stones

Skeletal specimen number	Teeth sampled	Age‐at‐death	Sex
WHIT 0219	LM1	40–60 years	Female
	LM3		
WHIT 0334	LM1	25–30 years	Male
	LM2		
WHIT 0559	LM2	30–40 years	Female
	LM3		

*Note*: Teeth sampled are all mandibular molars. Age‐at‐death estimations were provided by Andrew Chamberlain (pers. comm.). Sex determinations were established by Chamberlain and Witkin (2011; Chamberlain, pers. comm.), as well as Stewart et al. (2017) for WHIT 0219.

Age‐at‐death and sex estimations were established in the original osteological analysis by Chamberlain and Witkin (2011). Age‐at‐death was estimated using the Miles (1963) method, while sex estimations were based on the morphological characteristics of the mandible and, when possible, the cranium (Chamberlain & Witkin, 2011). Individual WHIT 0219 is also known to be biologically female based on sex‐specific isoforms of amelogenin (Stewart et al., 2017). Based on strontium (^87^Sr/^86^Sr) isotope results from a previous study by Neil et al. (2020) on tooth enamel, it is thought that the majority of the individuals interred at the Whitwell Long Cairn were migrants from continental Europe, potentially northwestern France. Of the individuals in the present study, WHIT 0219 and WHIT 0334 had ^87^Sr/^86^Sr ratios consistent with a childhood origin in continental Europe, while WHIT 0559 (referred to as WHIT 0512/2 in Neil et al. (2020)) had ^87^Sr/^86^Sr ratios compatible with a childhood origin in either Britain or continental Europe (Neil et al., 2020).

### 2.3 Sample preparation and stable isotope analysis

The teeth selected for analysis were first cleaned with acetone to remove any potential traces of PVA consolidant. The teeth were soaked in acetone and then set out to air dry to let the acetone evaporate. Once dry, teeth were rinsed with ultra‐purified water and set out to air dry again.

Using a dental saw, all teeth were bisected longitudinally. Four teeth were noted to have solid masses inside their pulp chambers. These masses, believed to be pulp stones, were left intact as they were adhered to the chamber walls. One half of each tooth was selected for sampling, and the enamel removed using a dental burr and saw, with the enamel‐dentine junction left intact when present. The roots of all samples were cleaned using a dental burr. For teeth without pulp stones, the pulp chamber was abraded with a dental burr to remove any secondary dentine.

Samples were demineralized in 0.5 M HCl at 4°C. Following the second method outlined in Beaumont et al. (2013), each tooth was cut horizontally into approximately one‐millimeter increments. For the teeth with pulp stones, the masses were removed using a scalpel. The edges of a pulp stone were first gently separated from the pulp chamber walls. The two separated easily once demineralized and no cutting was required. The scalpel was then inserted between the pulp stone and chamber wall at a slight angle, and the pulp stone lifted out by applying pressure beneath with the flat of the scalpel blade. Upon removal, the pulp stones were found to consist of several large and many smaller pieces. To be certain the smallest pieces were indeed removed, teeth were rinsed with ultra‐purified water to wash off any remaining pulp stone pieces. Dentine increments from the crowns of these teeth were also rinsed during sectioning to be certain no small pieces were clinging to them.

The dentine increments and a portion of each pulp stone were gelatinized in a pH 3 HCl acid solution heated at 70°C for 48 h. After heating, microtubes with any visible debris were centrifuged for 3 min at 5000 rpm. Samples were then frozen and freeze‐dried.

Stable isotope analysis was conducted following the methods and instrumentation outlined in Moore et al. (2020). The analytical uncertainty of carbon and nitrogen isotope ratios for replications of international standards was typically ±0.1‰. Duplicates of most samples were not run, as any potentially false results would be identifiable as outliers within the incremental dentine series.

### 2.4 Age calculations for incremental dentine samples

Age ranges were assigned to each dentine increment following Beaumont and Montgomery (2015). As the sections were approximately equal in size, they are tentatively assumed to represent growth periods of similar length, with the isotopic results for each increment reflecting a rolling average of a person's diet due to the appositional growth of dentine (Beaumont et al., 2013; Beaumont & Montgomery, 2015, 2016). The average developmental timespans for first, second, and third mandibular molars were drawn from the Queen Mary University of London (QMUL) Atlas of Human Development and Eruption by AlQahtani et al. (2010). Temporal ranges for each type of tooth were calculated by taking the median age at which a given tooth begins forming, and subtracting this number from the median age that particular tooth finishes forming. For each tooth sampled, its temporal range was divided by the total number of sections created during sampling to calculate the approximate amount of time each increment took to develop (Beaumont & Montgomery, 2015). As such, the estimated age calculation for any particular increment will vary based on the type and size of the tooth in question—teeth with a greater crown to root apex length will result in more dentine increments, which in turn will result in narrower time spans being calculated per incremental section. For the individuals in the present study, the time spans per increment typically range from about 7–11 months. For teeth with extensive occlusal wear, the corrective modeling method outlined in Halldórsdóttir et al. (2019) was used to make the time spans per increment more accurate. Increments were then modeled into a series by finding the mid‐points of their estimated growth periods, which were calculated by dividing the time span of each increment in half (Beaumont & Montgomery, 2015).

## 3 RESULTS

Collagen of sufficient preservation quality was recovered from both the incremental dentine and pulp stone samples. All teeth sampled had a collagen yield greater than 1%, and all dentine and pulp stone samples produced C:N ratios within the range of 3.1 and 3.5 (van Klinken, 1999). Two samples (5340‐1 and 5340‐2) were re‐analyzed to confirm their results, with the mean of the two analyses used hereafter. δ^13^C and δ^15^N values and collagen quality indicators for all dentine and pulp stone samples are presented in Table 2. Excluding the pulp stones, the group mean dentine δ^13^C value was −21.5 ± 0.2‰ and the mean δ^15^N value was 9.9 ± 0.5‰.

**TABLE 2:** Collagen carbon and nitrogen isotope results and quality indicators for the incremental dentine and pulp stone samples

Skeletal specimen number	Tooth	Laboratory code	Material	δ^13^C (‰)	δ^15^N (‰)	%C	%N	C:N atomic	Collagen yield (%)
**WHIT 0219**	**Left Molar 1**	5340‐1	Pulp stone	−21.6	9.2	42.3	15.1	3.3	
				−21.4	9.6	42.4	15.1	3.3	
			*Mean*:	*−21.5*	*9.4*				
		5340‐2	Dentine	−21.5	11.2	42.2	15.6	3.2	
				−21.4	11.3	42.1	15.2	3.2	
			*Mean*:	*−21.5*	*11.2*				
		5340‐3	Dentine	−21.4	10.0	41.5	15.2	3.2	
		5340‐4	Dentine	−21.3	9.7	42.1	15.5	3.2	
		5340‐5	Dentine	−21.6	9.8	42.2	15.4	3.2	
		5340‐6	Dentine	−21.6	9.9	42.2	15.4	3.2	
		5340‐7	Dentine	−21.4	10.4	42.9	15.6	3.2	
		5340‐8	Dentine	−21.6	10.1	42.6	15.4	3.2	
		5340‐9	Dentine	−21.8	10.1	42.5	15.2	3.3	
		5340‐10	Dentine	−22.1	9.8	42.5	15.1	3.3	
		5340‐11	Dentine	−21.9	10.1	42.8	15.2	3.3	
		5340‐12	Dentine	−21.6	10.1	42.5	15.0	3.3	
		5340‐13	Dentine	−21.7	10.1	42.5	14.7	3.4	16.8
**WHIT 0219**	**Left Molar 3**	5341‐1	Dentine	−21.3	9.3	42.9	15.5	3.2	
		5341‐2	Dentine	−21.0	8.9	42.8	15.4	3.2	
		5341‐3	Dentine	−20.9	9.0	43.1	15.5	3.2	
		5341‐4	Dentine	−21.1	9.7	43.2	15.5	3.2	
		5341‐5	Dentine	−21.2	9.8	42.7	15.2	3.3	
		5341‐6	Dentine	−21.3	10.2	43.6	15.6	3.3	
		5341‐7	Dentine	−21.5	10.2	42.5	15.2	3.3	
		5341‐8	Dentine	−21.3	10.6	42.7	15.4	3.2	
		5341‐9	Dentine	−21.5	9.9	42.4	15.1	3.3	
		5341‐10	Dentine	−21.5	9.7	42.4	15.1	3.3	
		5341‐11	Dentine	−21.4	10.1	42.8	15.2	3.3	
		5341‐12	Dentine	−21.4	10.1	42.9	15.1	3.3	
		5341‐13	Dentine	−21.6	10.3	43.4	15.2	3.3	
		5341‐14	Dentine	−21.7	10.1	43.6	15.1	3.4	17.8
**WHIT 0334**	**Left Molar 1**	5342‐1	Pulp stone	−21.5	9.6	43.0	15.3	3.3	
		5342‐2	Dentine	−21.8	10.0	42.8	15.3	3.3	
		5342‐3	Dentine	−21.5	9.2	43.2	15.5	3.2	
		5342‐4	Dentine	−21.6	9.1	43.4	15.6	3.3	
		5342‐5	Dentine	−21.8	9.2	42.9	15.4	3.2	
		5342‐6	Dentine	−21.7	9.5	42.8	15.4	3.2	
		5342‐7	Dentine	−21.8	9.5	42.6	15.2	3.3	
		5342‐8	Dentine	−21.7	9.4	42.5	15.3	3.3	
		5342‐9	Dentine	−21.6	9.3	42.7	15.3	3.3	
		5342‐10	Dentine	−21.7	9.2	42.7	15.2	3.3	
		5342‐11	Dentine	−21.8	9.9	42.5	15.2	3.3	
		5342‐12	Dentine	−21.5	10.4	42.8	15.2	3.3	
		5342‐13	Dentine	−21.6	10.3	42.7	15.0	3.3	
		5342‐14	Dentine	−21.7	9.9	43.5	15.2	3.3	
		5342‐15	Dentine	−21.2	10.3	42.4	15.1	3.3	18.0
**WHIT 0334**	**Left Molar 2**	5343‐1	Pulp stone	−21.4	9.8	42.8	15.1	3.3	
		5343‐2	Dentine	−22.2	9.4	42.8	15.2	3.3	
		5343‐3	Dentine	−21.9	9.7	43.3	15.6	3.2	
		5343‐4	Dentine	−21.7	9.1	42.4	15.3	3.2	
		5343‐5	Dentine	−21.6	9.3	42.6	15.3	3.2	
		5343‐6	Dentine	−21.6	9.8	42.5	15.3	3.2	
		5343‐7	Dentine	−21.5	9.9	42.7	15.4	3.2	
		5343‐8	Dentine	−21.6	9.5	43.4	15.6	3.2	
		5343‐9	Dentine	−21.6	9.4	43.2	15.5	3.2	
		5343‐10	Dentine	−21.7	9.3	43.0	15.4	3.2	
		5343‐11	Dentine	−21.7	9.2	42.2	15.2	3.2	
		5343‐12	Dentine	−21.6	8.8	43.0	15.4	3.3	
		5343‐13	Dentine	−21.7	9.2	42.7	15.2	3.3	
		5343‐14	Dentine	−21.6	10.1	42.6	15.2	3.3	
		5343‐15	Dentine	−21.6	10.6	42.6	15.1	3.3	
		5343‐16	Dentine	−21.5	10.7	42.8	15.2	3.3	
		5343‐17	Dentine	−21.5	10.3	42.4	14.9	3.3	
		5343‐18	Dentine	−21.6	10.0	41.7	14.5	3.4	17.1
**WHIT 0559**	**Left Molar 2**	5344‐1	Pulp stone	−21.5	9.1	42.7	14.7	3.4	
		5344‐2	Dentine	−21.5	10.6	42.8	15.4	3.2	
		5344‐3	Dentine	−21.4	10.7	42.8	15.3	3.3	
		5344‐4	Dentine	−21.5	10.5	43.0	15.5	3.2	
		5344‐5	Dentine	−21.3	10.1	42.9	15.4	3.3	
		5344‐6	Dentine	−21.1	10.3	42.8	15.4	3.2	
		5344‐7	Dentine	−21.2	10.5	42.8	15.3	3.3	
		5344‐8	Dentine	−21.1	10.4	43.0	15.4	3.3	
		5344‐9	Dentine	−21.2	10.3	43.3	15.3	3.3	
		5344‐10	Dentine	−21.3	10.4	43.1	15.3	3.3	
		5344‐11	Dentine	−21.3	10.6	42.8	15.2	3.3	
		5344‐12	Dentine	−21.3	10.7	42.8	15.2	3.3	
		5344‐13	Dentine	−21.4	10.8	42.5	15.0	3.3	
		5344‐14	Dentine	−21.2	10.6	42.7	15.0	3.3	
		5344‐15	Dentine	−21.4	10.4	42.4	14.8	3.4	
		5344‐16	Dentine	−21.7	10.4	42.7	14.6	3.4	15.2
**WHIT 0559**	**Left Molar 3**	5345‐1	Dentine	−21.2	10.9	42.7	15.3	3.2	
		5345‐2	Dentine	−21.2	10.7	42.9	15.5	3.2	
		5345‐3	Dentine	−21.1	10.6	42.7	15.4	3.2	
		5345‐4	Dentine	−21.0	10.1	42.7	15.3	3.2	
		5345‐5	Dentine	−21.2	9.9	42.8	15.4	3.2	
		5345‐6	Dentine	−21.5	9.4	42.4	15.2	3.3	
		5345‐7	Dentine	−21.5	9.6	42.6	15.2	3.3	
		5345‐8	Dentine	−21.6	9.8	42.7	15.2	3.3	
		5345‐9	Dentine	−21.7	9.8	42.8	15.3	3.3	
		5345‐10	Dentine	−21.9	9.2	42.4	15.0	3.3	
		5345‐11	Dentine	−21.7	9.1	42.5	15.2	3.3	
		5345‐12	Dentine	−21.5	9.2	42.6	15.1	3.3	
		5345‐13	Dentine	−21.3	9.6	41.8	15.3	3.2	
		5345‐14	Dentine	−21.6	10.0	41.8	15.2	3.2	
		5345‐15	Dentine	−21.8	10.3	41.9	15.1	3.2	
		5345‐16	Dentine	−21.6	10.3	42.0	15.0	3.3	15.5

*Note*: Collagen yields are calculated as the whole tooth sampled.

## 4 DISCUSSION

All individuals appear to have had terrestrial‐based diets, as collagen δ^13^C values below −20‰ for skeletal assemblages from north‐western Europe are thought to be indicative of diets in which most, if not all, of food was sourced from terrestrial plants and animals (Bonsall et al., 2009). While diets with a slight contribution (e.g., 10%) of protein from marine bivalves could produce collagen δ^13^C values around −20‰ (Bonsall et al., 2009), any regular consumption of marine bivalves is unlikely for the Whitwell individuals given the group mean δ^13^C value of −21.5 ± 0.2‰.

No faunal or botanical remains were available for the present study, so a local comparative baseline could not be established at this time. However, since at least some of the individuals from the cairn were probably migrants from continental Europe (Neil et al., 2020), a baseline comprising faunal and botanical remains from the cairn may not actually best reflect the environmental conditions of their residence during childhood and adolescence. Nonetheless, comparisons with isotopic data from contemporary Early Neolithic faunal remains from the Coneybury “Anomaly” (Gron et al., 2018) and the Ascott‐under‐Wychwood long barrow (Hedges, Stevens, & Pearson, 2007) in southern Britain suggest that the Whitwell individuals were a trophic level above herbivores such as sheep and cattle. Although these faunal remains are not ideal for comparison with the Whitwell individuals as there could be underlying factors that influenced their δ^13^C and δ^15^N values—such as differences in regional environmental conditions or distinct animal husbandry practices between groups—their comparison does suggest that the Whitwell individuals were gaining much of their dietary protein from meat and/or dairy products during childhood and adolescence. More detailed dietary reconstructions at the individual level are beyond the focus of the present study, but will be reported elsewhere.

The results from the pulp stones demonstrate that it is possible to obtain δ^13^C and δ^15^N values from pulp stone collagen, and show that sufficient quantities of collagen for stable isotope analysis can be obtained from just a portion of a pulp stone. Interpreting the results from the pulp stones, however, is a more complicated matter. The primary challenge is that it is difficult to establish when, and for how long, they formed during an individual's life. Another challenge is establishing if the pulp stone δ^13^C and δ^15^N values solely represent dietary practices or if other factors, like environmental conditions or an individual's health status, might be reflected in the results. An additional factor to consider is if there are any apparent conditions which might have caused, or at least contributed to, the formation of the pulp stones. Each of these factors are considered in turn to better assess the utility of pulp stones as a source of isotopic dietary information.

### 4.1 Can timeframes be established for the pulp stones?

To further investigate when the pulp stones might have formed, we compared their δ^13^C and δ^15^N results with the incremental dentine results for each individual. Since these profiles represent an individual's life from childhood through adolescence, identifying any similarities or differences between the incremental dentine and pulp stone δ^13^C and δ^15^N values could offer insight on when the pulp stones started forming.

#### 4.1.1 WHIT 0219

**FIGURE 3:**
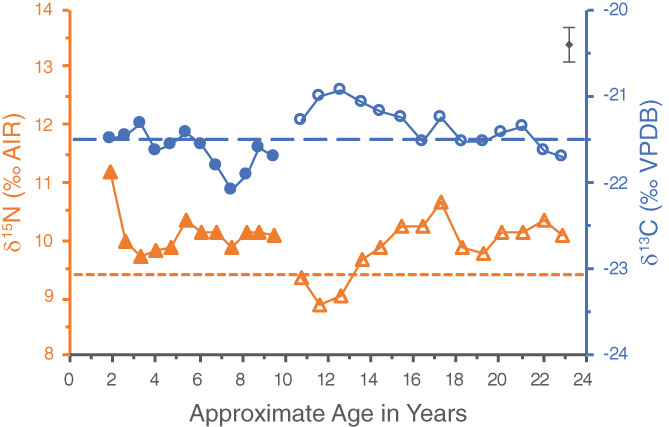
Incremental dentine series of WHIT 0219 comprised of the left first (closed symbols) and third (open symbols) mandibular molars. Circles represent δ^13^C values, whereas triangles represent δ^15^N values. The pulp stone, represented by the dashed lines, is from the first molar. The wider dashed line represents the pulp stone δ^13^C value, and the narrower dashed line represents its δ^15^N value. Analytical uncertainty of ±0.2‰ (2σ) is in the upper right corner

Individual WHIT 0219 had one pulp stone located in their first molar. The δ^15^N value of the pulp stone is 9.4‰—lower than all the increments of this particular tooth (see Figure 3). As such, the pulp stone presumably did not begin forming until after this molar finished developing. The pulp stone δ^15^N value could perhaps represent an average of the δ^15^N values of the third molar, though in this scenario the pulp stone would have had to begin forming before the age of 14 for its δ^15^N value to be lower than the remainder of the third molar series. This does not, however, fit well with the pulp stone δ^13^C value of −21.5‰, as this value is lower than those of all the third molar dentine increments until age 16. Given this, it seems most likely that the pulp stone formed after both the first and third molars finished developing. The pulp stone may have formed over quite a number of years, as the median age at which third molars typically finish developing is 23.5 ± 0.5 years (AlQahtani et al., 2010), and the estimated age‐at‐death for WHIT 0219 is 40–60 years. Moreover, this tooth was severely worn with the occlusal enamel and first few millimeters of dentine missing. The pulp stone may have formed over time in response to this progressing dental attrition.

If the pulp stone did form after this individual's teeth finished developing, its δ^13^C and δ^15^N values would reflect diet after the timeframe of the dentine series. While the δ^13^C value of the pulp stone is consistent with those of the last few dentine increments, its δ^15^N value represents a significant drop in comparison (Table 2 and Figure 3). A possible explanation for why the δ^15^N value of the pulp stone is lower than those of the last dentine increments could be a change in diet and/or health of this individual in later life.

#### 4.1.2 WHIT 0334

**FIGURE 4:**
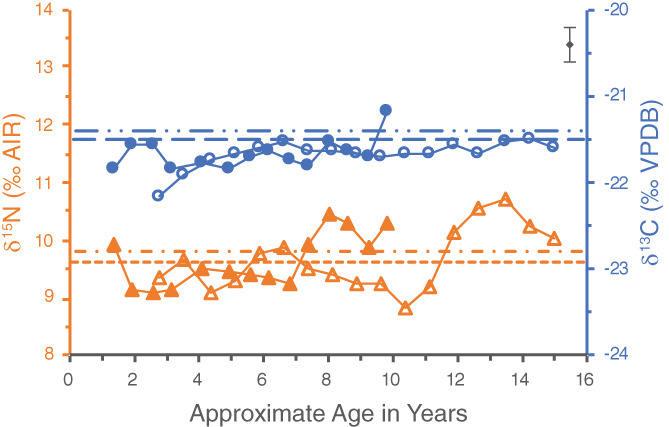
Original incremental dentine series of WHIT 0334 comprised of the left first (closed symbols) and second (open symbols) mandibular molars. Circles represent δ^13^C values, whereas triangles represent δ^15^N values. The first molar pulp stone is represented by the regular dashed lines, with the wider dashed line designating its δ^13^C value and the narrower dashed line its δ^15^N value. The second molar pulp stone is represented by the dashed and dotted lines, with the δ^13^C value designated by the line with one dot between dashes, and the δ^15^N value by the line with two dots between dashes. Analytical uncertainty of ±0.2‰ (2σ) is in the upper right corner

**FIGURE 5:**
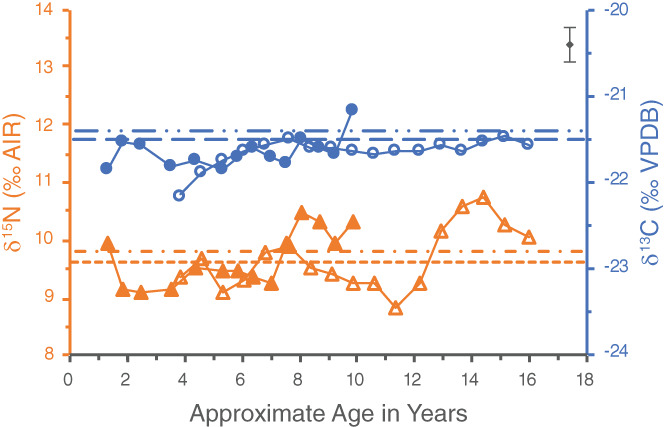
“Wiggle‐matched” incremental dentine series of WHIT 0334 comprised of the left first (closed symbols) and second (open symbols) mandibular molars. Circles represent δ^13^C values, whereas triangles represent δ^15^N values. The first molar pulp stone is represented by the regular dashed lines, with the wider dashed line designating its δ^13^C value and the narrower dashed line its δ^15^N value. The second molar pulp stone is represented by the dashed and dotted lines, with the δ^13^C value designated by the line with one dot between dashes, and the δ^15^N value by the line with two dots between dashes. Analytical uncertainty of ±0.2‰ (2σ) is in the upper right corner

The dentine series for WHIT 334 appears to be misaligned slightly between the first and second molars, most visible in the offset of the similar δ^15^N patterns of both teeth (Figure 4). This may be due to differences in resolution—the first molar was particularly difficult to section and the timeline for the increments had to be adjusted slightly to accommodate one thicker increment (sample 5342‐6). The offset could also result from using average developmental timelines based on the median age of tooth development for the molars, drawn from AlQahtani et al. (2010), which may not accurately reflect this individual's development. For instance, if the second molar began developing later in childhood, then the δ^15^N profiles between the two teeth align more closely (Figure 5).

A second profile was therefore created using the “wiggle‐matching” technique (Figure 5). In this technique, the age estimations of when particular teeth begin and finish developing are adjusted to align the isotopic profiles of co‐forming teeth (Beaumont, 2020). For some individuals, this method may make it possible to better account for individual variation in the timing of dental development. This technique was used in the analysis of incremental dentine from soldiers from the Battle of Dunbar (1650 AD), for example, which enabled researchers to better align a particular individual's stage of dental development with their estimated age‐at‐death in a number of instances (Beaumont, 2020; Millard et al., 2020).

The major adjustment in the wiggle‐matched profile for WHIT 0334 is that the age at which their second molar began forming has been pushed back to age 3.5, which results in the midpoint of the first increment of their second molar being about 3.9 years (Figure 5). Based on the assumption that the dentine increments formed over similar lengths of time, this in turn pushes the age at which the second molar finished developing to approximately age 16. While the dentine series of the two teeth still do not line up exactly, the adjustments made with the wiggle‐matching technique do appear to better align them. Interpretations for WHIT 0334 were therefore made based on the wiggle‐matched profile.

This individual had pulp stones in each of the teeth sampled, a first and second molar. The pulp stones had δ^13^C values of −21.5‰ and −21.4‰, and δ^15^N values of 9.3‰ and 9.8‰, respectively. The δ^15^N and δ^13^C values of the first molar pulp stone could feasibly represent an average of the values exhibited by the dentine increments in either the first or second molar. The δ^15^N and δ^13^C values of the second molar pulp stone could both be consistent with an average of the second molar dentine increments, particularly after age 6, as the δ^13^C values of the pulp stone and dentine increments are very comparable after this point. These results may tentatively suggest that this pulp stone began forming sometime after early childhood.

This person was the youngest of the individuals sampled, with an estimated age‐at‐death of 25–30 years. Although there was some exposed dentine on the occlusal surfaces of these teeth, particularly for the first molar, the wear of the second molar was not as advanced as the other teeth with pulp stones. Despite this, both pulp stones completely occluded their pulp chambers, suggesting they may have been forming for some time. It therefore seems unlikely they were forming solely in response to dental wear, and there may have been some other etiology or combination of factors influencing their development.

If these pulp stones did form after the molars finished developing, their δ^13^C values suggest that the adult diet and environmental conditions of this individual were similar to those of their childhood and adolescence. The differential δ^15^N values would likewise be similar to the variability in δ^15^N exhibited throughout the dentine increments, and may suggest this person's protein input and/or health status varied frequently throughout their life.

#### 4.1.3 WHIT 0559

**FIGURE 6:**
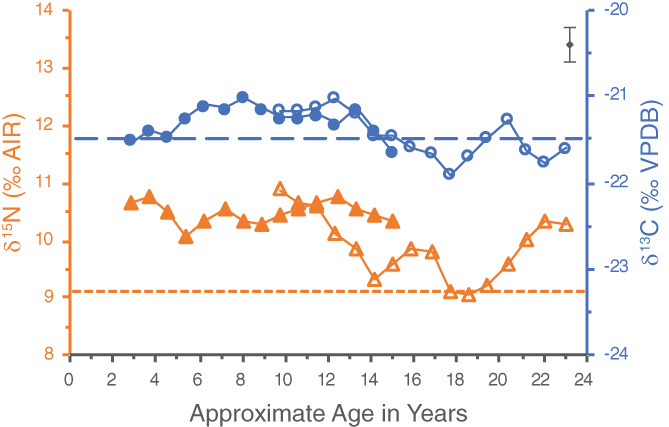
Original incremental dentine series of WHIT 0559 comprised of the left second (closed symbols) and third (open symbols) mandibular molars. Circles represent δ^13^C values, whereas triangles represent δ^15^N values. The pulp stone, represented by dashed lines, is from the second molar. The wider dashed line represents the pulp stone δ^13^C value, and the narrower dashed line represents its δ^15^N value. Analytical uncertainty of ±0.2‰ (2σ) is in the upper right corner

The initial dentine series for WHIT 0559 also appears to be misaligned slightly (Figure 6). The molar timelines were created using the median ages of development from AlQahtani et al. (2010), but as the timing of the third molar is most variable between individuals, this may not be accurate for WHIT 0559. A later age for this molar's formation would better account for the area of overlap with the first molar in Figure 6.

**FIGURE 7:**
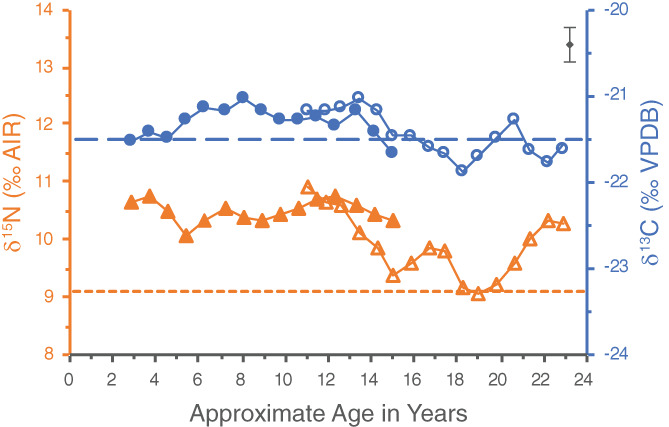
“Wiggle‐matched” incremental dentine series of WHIT 0559 comprised of the left second (closed symbols) and third (open symbols) mandibular molars. Circles represent δ^13^C values, whereas triangles represent δ^15^N values. The pulp stone, represented by dashed lines, is from the second molar. The wider dashed line represents the pulp stone δ^13^C value, and the narrower dashed line represents its δ^15^N value. Analytical uncertainty of ±0.2‰ (2σ) is in the upper right corner

An additional wiggle‐matched profile was therefore created for WHIT 0559 to better align their dentine series (Figure 7). The start of the third molar's development has been pushed back to age 10, which makes the midpoint of its first increment approximately age 11. The end of the third molar's development has been left at 23.5 years of age, as a condensed timeline for this molar seems to best align the two teeth.

This individual had one pulp stone located in their second molar. The pulp stone had a δ^13^C value of −21.5‰ and a δ^15^N value of 9.1‰. This δ^15^N value is significantly lower than all of the increments from the second molar, suggesting the pulp stone did not form while this tooth was developing. The third molar, however, exhibits δ^15^N values similar to that of the pulp stone around ages 18–20. The δ^13^C value of the pulp stone is not drastically different from those of the dentine increments at this age, so it could be feasible that the pulp stone formed at this time in the individual's life. This pulp stone was also significantly smaller than the others and was not completely occluding the chamber, which could present two scenarios: the pulp stone formed at this time and did not grow any further; or the pulp stone formed later in life and had not been developing for a long enough period to grow to a significant size before this individual's death. If the latter scenario is correct, the pulp stone could have formed at any time after this individual's third molar finished developing and before their death, estimated at 30–40 years old.

If the pulp stone did form during adulthood, its δ^15^N value, which is significantly lower than the last increment of the third molar at 9.1‰ and 10.3‰, respectively, could represent some dietary change in this person's adult life. A possible explanation could be a reduction in the proportion of dietary protein coming from animals as opposed to plants.

### 4.2 Are the pulp stone results suggestive of particular dietary practices?

The possible link between pulp stone formation and diets high in animal protein proposed by Nicklisch et al. (2021) is intriguing for the present study, as it does seem likely that the Whitwell individuals were consuming meat and/or dairy products during their childhood and adolescence. The δ^15^N values of the pulp stones from WHIT 0219 and WHIT 0559, however, are lower than the δ^15^N values of most of their dentine increments, which may suggest that the pulp stones of these two individuals did not actually form when their diets were particularly high in animal protein. This is complicated, however, by the influence of physiological stress on δ^15^N values. During periods of physiological stress, catabolism can occur, in which the body recycles its own tissues to release stored nutrients. This results in an increase in δ^15^N as a trophic level effect occurs within a person's body (Mekota et al., 2006, 2009). Catabolism can also produce a decrease in δ^13^C values through the recycling of stored body fat, which has lower δ^13^C values than other body tissues (Neuberger et al., 2013; Tieszen & Fagre, 1993). In the profile of WHIT 0219, there does appear to be a possible catabolic effect occurring from about age 12 to 17.5 (Figure 3), where there is a significant and prolonged increase in δ^15^N accompanied by a slight drop in δ^13^C values. Interpretations regarding dietary protein and pulp stone formation must therefore be made cautiously, as δ^15^N values may not simply reflect an individual's trophic level. Future isotopic research on the potential correlation between pulp stone prevalence and dietary protein should include comparative faunal and botanical baselines, and might also compare δ^15^N values of pulp stones, dentine, and other skeletal elements with different collagen turnover rates from the same individual to better understand an individual's diet over time.

### 4.3 Possible etiologies

**FIGURE 8:**
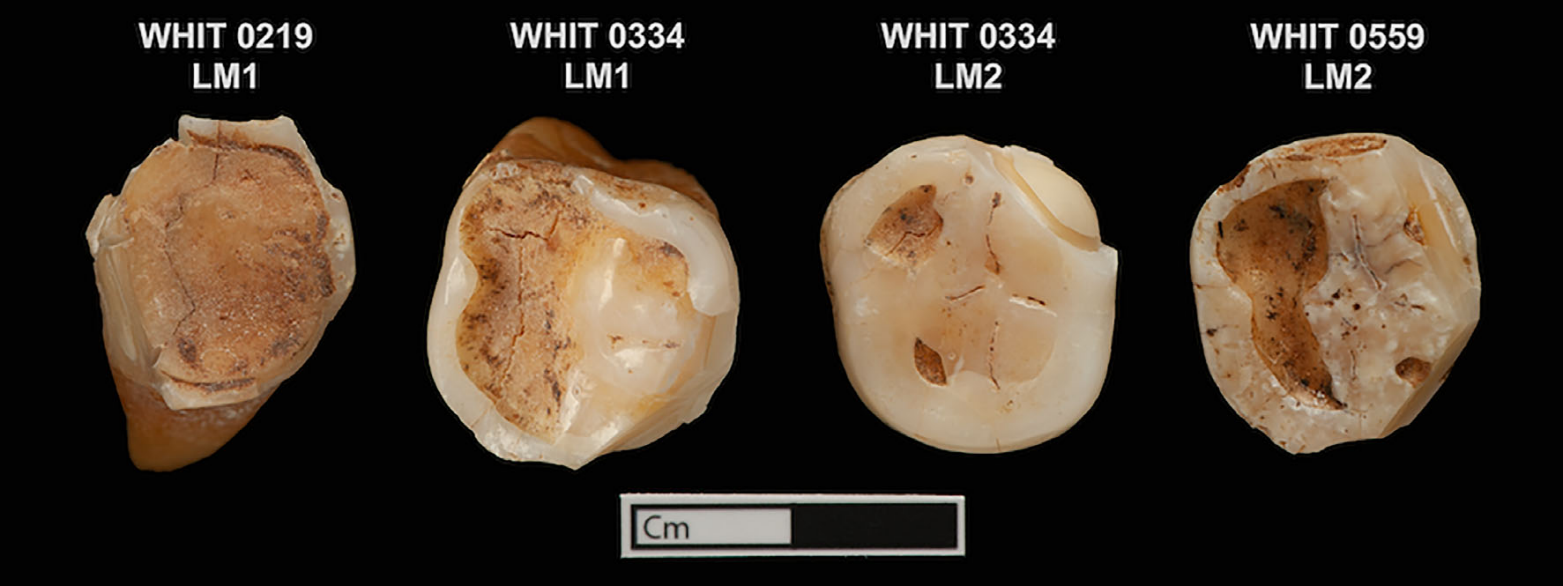
View of the occlusal surfaces of the teeth that contained pulp stones. All teeth have areas on the buccal or lingual sides were enamel was previously sampled for isotopic analysis. “LM1” designates the tooth is a left first molar. “LM2” designates the tooth is a left second molar. All teeth are mandibular molars

The difficulty in interpreting isotopic data from pulp stones is further compounded by the fact that it is not known why the pulp stones formed, which undoubtedly influenced the timing and duration of their development. A possible etiology for the individuals presented here could be advanced dental wear, which has been suggested to instigate pulp stone formation through long‐term irritation of the dentine and pulp (Tomczyk et al., 2014, 2017). Pulp stone prevalence has been found to have a correlation with dental wear in a number of archaeological and historical skeletal assemblages (Elvery et al., 1998; Tomczyk et al., 2014, 2017, 2020). In the present study, the teeth with pulps stones all had exposed dentine on their occlusal surfaces (Figure 8). The dental wear of the second molar of WHIT 0334, however, was slight compared to the other individuals, yet this individual had two pulp stones that were completely occluding the pulp chambers of the teeth examined. It may be that there was another factor contributing to the development of pulp stones for this skeletal assemblage, though dental wear likely also contributed to the formation of pulp stones for these individuals, particularly for WHIT 0219. Additional research, such as aDNA analysis, might be able to pinpoint other possible etiologies besides dental wear within the group, such as a genetic predisposition.

Future research could also consider the potential of environmental conditions as a contributing factor to pulp stone formation. Tomczyk et al. (2014, 2020) have proposed that there may be a connection between pulp stone prevalence and high levels of calcium in the diet. If pulp stone formation were influenced by dietary calcium intake, a similar environmental connection could be proposed for hard water consumption. For archeological skeletal assemblages, individuals excavated from regions of chalk and limestone tend to have low enamel strontium concentrations due in part to the high calcium levels present in both soils and waters in these regions. The range of strontium concentrations in Beaker period humans from southern Britain, for example, is 14–131 ppm, with those from chalk and limestone sites having strontium concentrations at the lower end of this range (Montgomery et al., 2019). Many of the individuals from the Whitwell Long Cairn also had low enamel strontium concentrations (Neil et al., 2020), including WHIT 0219 and WHIT 0334 from the present study, which might likewise suggest high levels of calcium in the water and soil of their childhood environments.

### 4.4 Assessing the usefulness of pulp stones for palaeodietary reconstructions

The comparisons presented here demonstrate that when the δ^13^C and δ^15^N values of pulp stones differ greatly from their respective incremental dentine series, they can potentially extend dietary information beyond childhood and adolescence. This would likely be most useful for palaeodietary studies conducted on fragmentary and comingled skeletal assemblages in which it may not be possible to match skeletal elements commonly used for investigating adult diet, such as the ribs, with the teeth of specific individuals. However, even when they seem to represent a dietary change later in adulthood, interpretations of isotopic data from pulp stones will be limited in that it will probably be impossible to suggest with certainty when exactly in adulthood they formed, or what length of time the data represents. Moreover, when their δ^13^C and δ^15^N values are similar to those of dentine increments, pulp stones will not add particularly useful palaeodietary information, as it will be difficult to determine when they formed relative to the tooth dentine during life. Such was the case for individual WHIT 0334 in the present study. The usefulness of paleodietary stable isotope information from pulp stones thus hinges on there being *a clear difference* between the δ^13^C and δ^15^N values of the pulp stones in question and the incremental dentine data of an individual's isotopic profile. We therefore recommend that stable isotope analysis of pulp stones only be undertaken when researchers are already employing the incremental dentine sampling strategy. Moreover, comparisons with incremental dentine could help identify patterns that might be indicative of poor health and/or nutritional stress, and thereby offer insight into factors that may have affected the δ^13^C and δ^15^N values of an individual's pulp stones. Given the challenges identified here, analyzing pulp stones on their own without incremental dentine is not recommended, especially as their analysis is not necessarily less destructive than sampling other skeletal tissues since a tooth needs to be sectioned for their extraction.

## 5 CONCLUSION

The method for extracting pulp stones from demineralized teeth samples presented here was simple to perform, required no additional equipment than that already needed for incremental dentine sectioning, and should be easy for other researchers to replicate. The results demonstrated it is possible to obtain palaeodietary information from pulp stones using stable isotope analysis. The usefulness of this information, however, will depend on the larger sampling strategy employed. It will likely be most relevant when already conducting incremental dentine sampling on the teeth in question, as comparisons of pulp stone δ^13^C and δ^15^N values with those from incremental dentine may provide indications of the timing of pulp stone formation, and may help identify other factors, such as nutritional stress, that could influence δ^13^C and δ^15^N values. Without incremental dentine results with which to compare, data from pulp stones alone will reflect an individual's average diet for an unknown duration of time from an unspecified period of their life, resulting in data with fairly limited value that is still destructive to obtain. In the present study, comparisons of data from pulp stones and incremental dentine made it possible to extend the life histories of two of the individuals examined beyond childhood and adolescence. It was not possible, however, to determine with certainty if the pulp stones of the third individual formed during or after the time period represented by their incremental dentine profile, further highlighting the difficulty of contextualizing isotopic results from pulp stones, even with accompanying incremental dentine data. All teeth with pulp stones exhibited moderate to severe occlusal wear, which may have contributed to the development of the pulp stones, though other unknown contributing factors cannot be discounted. Additional research on this skeletal assemblage, and on pulp stones more broadly, might further investigate the possible links between pulp stone formation and diet as proposed by other researchers, as well as consider health conditions or possible environmental factors, such as calcium intake from hard water consumption, that may influence pulp stone formation.

## CONFLICT OF INTEREST

The authors declare no conflicts of interest.

## AUTHOR CONTRIBUTIONS

**Brett Ostrum:** Conceptualization (equal); data curation (equal); formal analysis (equal); investigation (equal); methodology (equal); writing – original draft (lead); writing – review and editing (equal). **Darren R. Gröcke:** Data curation (equal); formal analysis (equal); funding acquisition (supporting); investigation (equal); methodology (equal); resources (equal); writing – review and editing (equal). **Janet Montgomery:** Conceptualization (equal); data curation (equal); formal analysis (equal); funding acquisition (lead); methodology (equal); project administration (lead); resources (equal); supervision (lead); writing – review and editing (equal).

## Data Availability

The data of this study is available within Table 2 of the article and will also be stored within the IsoArcH data repository (https://isoarch.eu).
